# The Histamine H3 Receptor Antagonist DL77 Ameliorates MK801-Induced Memory Deficits in Rats

**DOI:** 10.3389/fnins.2018.00042

**Published:** 2018-02-12

**Authors:** Nermin Eissa, Nadia Khan, Shreesh K. Ojha, Dorota Łazewska, Katarzyna Kieć-Kononowicz, Bassem Sadek

**Affiliations:** ^1^Department of Pharmacology & Therapeutics, College of Medicine & Health Sciences, United Arab Emirates University, Al Ain, United Arab Emirates; ^2^Department of Technology and Biotechnology of Drugs, Faculty of Pharmacy, Jagiellonian University-Medical College, Kraków, Poland

**Keywords:** histamine H3 receptor, antagonist, learning and memory, Alzheimer's disease, neurodegeneration, passive avoidance paradigm, novel object recognition, behavioral research

## Abstract

The role of Histamine H3 receptors (H3Rs) in memory, and the prospective of H3R antagonists in pharmacological control of neurodegenerative disorders, e.g., Alzheimer disease (AD) is well-accepted. For that reason, the procognitive effects of the H3R antagonist DL77 on cognitive impairments induced with MK801 were tested in an inhibitory passive avoidance paradigm (PAP) and novel object recognition (NOR) task in adult male rats, using donepezil (DOZ) as a standard drug. Acute systemic pretreatment with DL77 (2.5, 5, and 10 mg/kg, i.p.) significantly ameliorated memory deficits induced with MK801 in PAP (all *P* < 0.05, *n* = 7). The ameliorative effect of most promising dose of DL77 (5 mg/kg, i.p.) was reversed when rats were co-injected with the H3R agonist *R*-(α)-methylhistamine (RAMH, 10 mg/kg, i.p.) (*p* = 0.701 for MK801-amnesic group vs. MK801+DL77+RAMH group, *n* = 6). In the NOR paradigm, DL77 (5 mg/kg, i.p.) counteracted long-term memory (LTM) deficits induced with MK801 (*P* < 0.05, *n* = 6–8), and the DL77-provided effect was similar to that of DOZ (*p* = 0.788, *n* = 6–8), and was reversed when rats were co-injected with RAMH (10 mg/kg, i.p.) (*p* = 0.877, *n* = 6, as compared to the (MK801)-amnesic group). However, DL77 (5 mg/kg, i.p.) did not alter short-term memory (STM) impairment in NOR test (*p* = 0.772, *n* = 6–8, as compared to (MK801)-amnesic group). Moreover, DL77 (5 mg/kg) failed to modify anxiety and locomotor behaviors of animals innate to elevated-plus maze (EPM) (*p* = 0.67 for percentage of time spent exploring the open arms, *p* = 0.52 for number of entries into the open arms, *p* = 0.76 for percentage of entries into the open arms, and *p* = 0.73 number of closed arm entries as compared to saline-treated groups, all *n* = 6), demonstrating that the procognitive effects observed in PAP or NOR tests were unconnected to alterations in emotions or in natural locomotion of tested animals. These results signify the potential involvement of H3Rs in modulating neurotransmitters related to neurodegenerative disorders, e.g., AD.

## Introduction

Alzheimer's disease (AD) is a life long-lasting brain disorder that is considered by its cognitive deficits, memory impairment, and dementia (Khunnawutmanotham et al., 2016; Shaik et al., 2016). Following a recent report, around 36 million people worldwide were diagnosed with dementia in recent years, and the number is estimated to significantly increase by the double every two decades, ultimately leading to more than 100 million people with AD after four decades (Khunnawutmanotham et al., 2016). The pathogenesis of AD is still complex, though several theories have been established. The multifaceted pathophysiological alterations include deficient cholinergic neurotransmission, malfunctioning metabolic status of β-amyloid protein, irregularities of numerous central neurotransmitters including glutamate, norepinephrine, serotonin and dopamine, and the contribution of neuroinflammation and high oxidative stress to the progression of AD (Doraiswamy, 2002; Khan et al., 2015; Sadek et al., 2016a). Brain histamine is an established neurotransmitter in the central nervous system (CNS) (Arrang et al., 1983, 1985, 1987a,b, 1988, 2007; Schwartz et al., 1986), exerting its biological activities through interaction with four histamine receptor (HR) subtypes (H1-H4R) that belong to the family of G-protein coupled receptors (Schneider and Seifert, 2009; Panula et al., 2015). H1R and H2R are present in the brain and periphery, whereas H4Rs are predominately expressed in mast cells and leukocytes (Schneider and Seifert, 2009; Panula et al., 2015). Contrary, H3Rs are abundant in the CNS (Arrang et al., 1983, 1985, 1987a,b, 1988, 2007; Panula et al., 2015). Moreover, H3Rs acting as auto-receptors in the CNS are coupled to Gα_i/o_-proteins and are capable of controlling the synthesis and release of histamine (Arrang et al., 1983, 1985, 1987a,b, 1988, 2007). Furthermore, H3Rs operating as hetero-receptors located on non-histaminergic neurons in different brain regions can also moderate the release of other neurotransmitters including acetylcholine, glutamate, GABA, norepinephrine, serotonin, dopamine (Brown et al., 2001, 2013). Previous preclinical experiments have proposed H3R antagonists to be of characteristic feature by their possible memory-enhancing effects (Panula et al., 1998, 2015; Panula and Nuutinen, 2011; Sadek and Stark, 2015; Sadek et al., 2016b). Accordingly, several H3R antagonists improved cognitive deficits induced with ketamine and MK801 in numerous animal models (Browman et al., 2004), suggesting that these H3R antagonists may also be effective in neurodegenerative disorders, e.g., AD (Witkin and Nelson, 2004; Bardgett et al., 2010; Charlier et al., 2013; Sadek and Stark, 2015; Sadek et al., 2016b). Among a wide range of H3R antagonists investigated so far, H3R antagonists ABT-239 and A-431404 were found to ameliorate cognitive deficits induced by ketamine and MK-801 in rodents, demonstrating enhanced procognitive effects of these compounds compared to standard drugs, e.g., DOZ (Brown et al., 2013). Therefore, the central H3Rs embodies an attractive target for the development of novel H3R antagonists with the prospective therapeutic future in neurodegenerative disorders (Yokoyama et al., 1993; Yokoyama, 2001; Harada et al., 2004; Witkin and Nelson, 2004; Uma Devi et al., 2010; Bhowmik et al., 2012, 2014; Sadek and Stark, 2015; Sadek et al., 2016b). Regardless of the abovementioned experimental observations for the role for H3Rs in the modulation of memory deficits and related behaviors, targeting H3Rs in the CNS is not commonly proposed as a future strategy to treat AD.

In the current study and as a continuation of our research efforts, the procognitive effects of the non-imidazole H3R antagonist, namely DL77 [1-(3-(4-*tert*-pentylphenoxy)propyl)piperidine dihydrogenoxalate], with high *in vitro* selectivity to human H3R, high antagonist affinity in the subnanomolar concentration range and a p*K*_i_-value of 8.03, and high H3R antagonist *in vivo* potency with an ED_50_ value of 2.1 ± 0.2 mg/kg, per oral (p.o.) (Łazewska et al., 2006) has been explored on its procognitive effects on memory deficits induced with MK801 in PAP and NOR paradigms in adult male rats applying DOZ as a reference drug. Moreover, the effects of DL77 on anxiety and locomotor behaviors in EPM was assessed, since anxiety and locomotion could influence the performance of rats in PAP or NOR. Furthermore, the abrogative effects of the histamine H3R agonist RAMH on the memory-enhancing effects provided with DL77 in PAP and NOR paradigms were examined. The non-imidazole H3R antagonist DL77 was selected for testing in the current studies as it in an earlier study proved to exhibit (5–15 mg/kg, i.p.) anticonvulsant properties and procognitive effects on acquisition, consolidation and retrieval in the same animal species (Sadek et al., 2016c). Also, a previous study showed a promising effects of DL77 (3–30 mg/kg, i.p.) on alcohol intake and preference in adult C57BL/6 mice (Bahi et al., 2015).

## Experimental procedures

### Animals

Male Wistar rats (inbred of Central Animal Facility of the UAE University, aged 6–8 weeks) of body weight 180–220 g were used for the study. The animals were retained in an air-conditioned animal facility room with controlled temperature (24 ± 2°C) and humidity (55 ± 15%) under a 12 h light/dark cycle, and were provided free access to food and water. Experiments were carried out between 9:00 and 13:00 h, and all procedures were approved by the Institutional Animal Ethics Committee of College of Medicine and Health Sciences/United Arab Emirates University (A30-13). All efforts were made to minimize animal suffering, to reduce the number of animals used. Also, all behavioral studies were conducted in a blinded fashion and by the same experimenter.

### Drugs

*R*-(α)-methylhistamine dihydrochloride (RAMH), donepezil hydrochloride (DOZ), and MK801 hydrogen maleate were purchased from Sigma-Aldrich (St Louis, Missouri, USA). The H3R antagonist 1-(3-(4-*tert*-pentylphenoxy)propyl)piperidine dihydrogenoxalate (DL77, 2.5, 5, 10, mg/kg) was synthesized by us in the Department of Technology and Biotechnology of Drugs (Kraków, Poland) as described previously (Meier et al., 2001; Łazewska et al., 2006). All drugs were dissolved in isotonic saline and injected intraperitoneal (i.p.) at a volume of 1 ml/kg, and all doses were expressed in terms of the free base.

### Behavioral tests

#### Inhibitory PAP test

Male adult Wistar rats were tested in a two compartment step-through passive avoidance apparatus (Step-through Cage, 7550, Ugo Basile, Comerio, Italy) as described previously (Izquierdo et al., 1999; Bernaerts et al., 2004; da Silva et al., 2009; Goshadrou et al., 2013; Khan et al., 2015; Sadek et al., 2016a,c; Alachkar et al., 2017; Sultan et al., 2017), with minor modifications. The test was conducted in an automatically operated commercial passive avoidance apparatus as previously described (Khan et al., 2015; Sadek et al., 2016a,c; Alachkar et al., 2017). The experiment consisted of two trials (training and testing) separated by a 24 h interval. Each rat in the first trial was placed in the white compartment (facing the auto guillotine door) and after a 30-s habituation period the door was raised automatically. The rat was given a 60 s cut-off time to step-through to the dark compartment. Rats that failed to move within this time period were excluded from the test session on the following day. Once the rat moved into the dark compartment, the sliding door was lowered and a scrambled foot shock (0.4 mA, 20 Hz, 8.3 ms) was delivered to the grid floor for a duration of 3 s. The power of the delivered foots-hock was designated following confirming the sensitivity threshold that yields the minimal vocalization and jumping responses in tested rats. The rat was removed from the dark chamber directly after receiving the foot-shock, returned to its home cage, and both compartments were cleaned. The animals were trained for 3 consecutive days; in which they were injected with saline i.p. 30–45 min before each training session, with the only modification that the cut-off latency was put at 300 s to move to the dark compartment without delivery of scrambled foot-shock (Khan et al., 2015; Sadek et al., 2016a,c). Rats that did not move into the dark compartment during the training, despite the practices carried out during training sessions, were excluded from the current test. For each separate experiment, 9–11 animals of the same age and weight average were trained on the step-through latency (STL) paradigm. Approximately 2–4 rats failed to demonstrate enhanced performance in a cut-off time of 60 s. In the current series of behavioral experiments, a group of 7 animals was used for each STL task conducted for the PAP. In the test session, animals were turned amnesic with acute systemic injection of MK801 (0.1 mg/kg, i.p.) 30–45 min prior to test session, and the animals were allowed to move to the dark compartment for a maximum period of 300 s. In this test session, the STL time for each rat to enter the dark compartment in 300 s was measured.

##### Dose regimen

**Figure 1:**
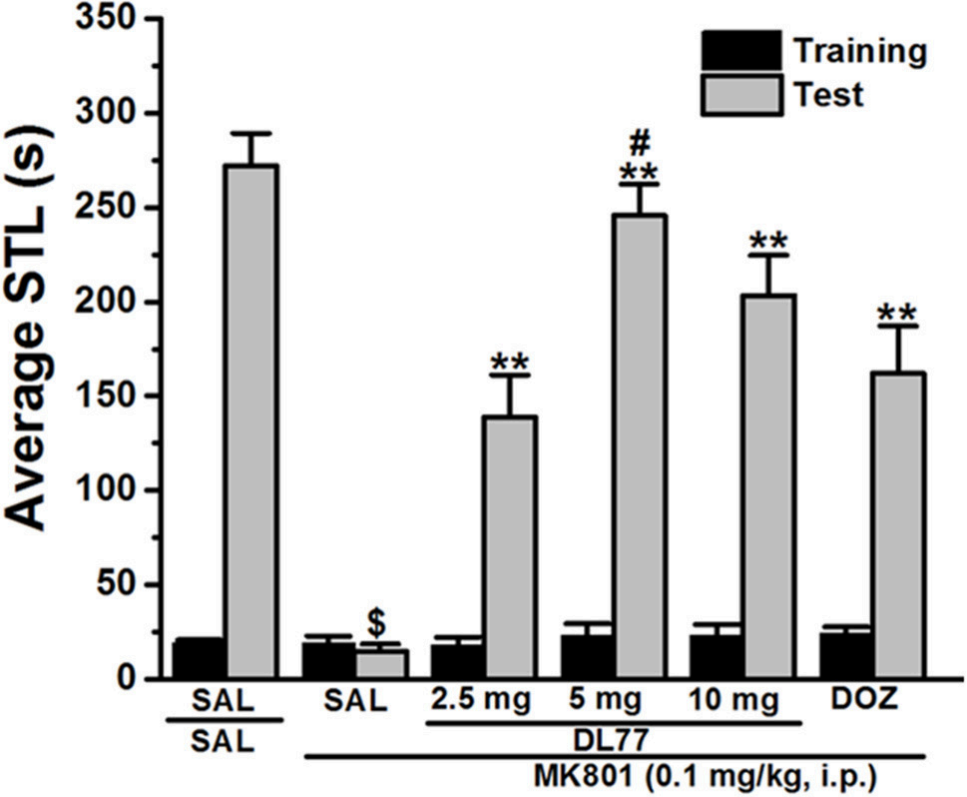
Effects of DL77 on MK801-induced memory deficits in an inhibitory PAP in rats. Gray columns represent the mean STLs measured during the training trial before the delivery of the foot-shock (pre-shock latencies) and black columns the mean STLs measured during the retention test (test latencies). Rats were injected with DL77 (2.5, 5, or 10 mg/kg, i.p.) or donepezil (DOZ, 1 mg/kg, i.p.) 30 min before the test session. ^$^*P* < 0.001 for mean STLs vs the value of the (saline)-treated group. ^**^P < 0.001 for mean STLs vs. the value of the (MK801)-treated group. ^#^*P* < 0.05 for mean STLs vs. the value of MK801+DOZ-treated group. Data are expressed as mean ± SEM (*n* = 7).

**Figure 2:**
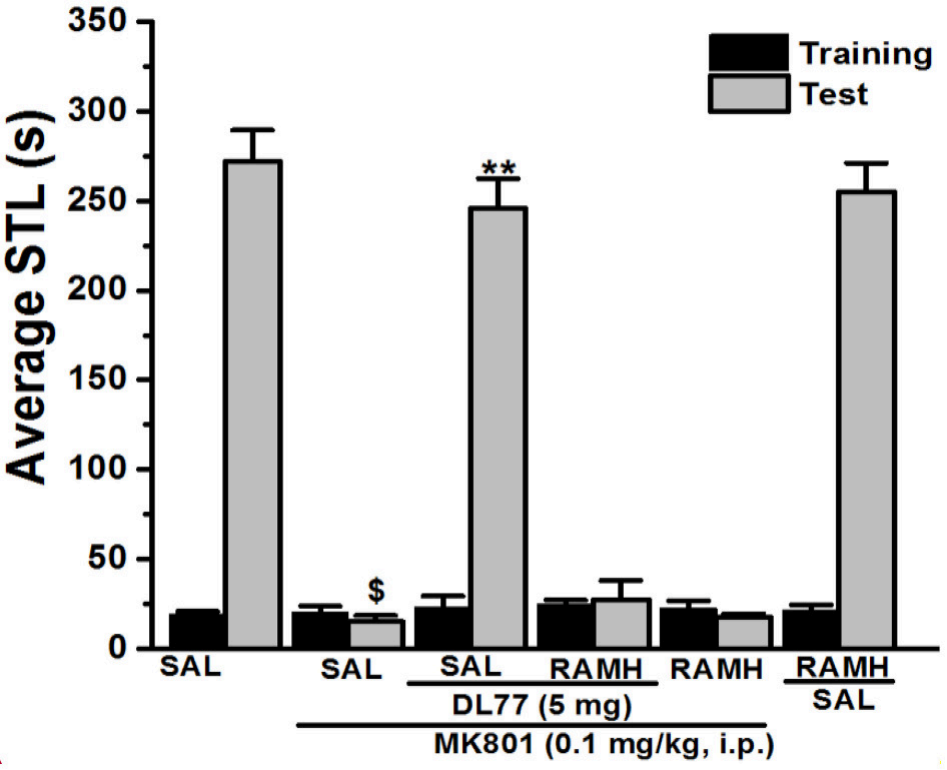
Effect of vehicle, DL77, and RAMH on MK801-induced deficit in an inhibitory PAP in rats. Gray columns represent the mean STLs measured during the training trial before the delivery of the foot-shock (pre-shock latencies) and black columns the mean STLs measured during the retention test (test latencies). Rats were injected with DL77 (5 mg/kg, i.p.), RAMH, or a combination of RAMH (10 mg/kg)+DL77 (5 mg/kg) 15 min (RAMH) or 30 min (DL77) before the test session. ^$^*P* < 0.001 for mean STLs vs. the value of the (Saline)-treated group. ^**^P < 0.001 for mean STLs vs. the value of the (MK801)-treated group. Data are expressed as mean ± SEM (*n* = 7).

Six groups of seven rats each were used, and were pretreated with Saline+Saline, MK801+Saline, MK801+DL77 (2.5, 5, and 10 mg/kg, i.p.), or MK801+DOZ (1 mg/kg, i.p.) 30–45 min before the test session, and the procognitive effects DL77 (2.5, 5, and 10 mg/kg, i.p.) on amnesia induced with acute systemic injection of MK801 (0.1 mg/kg, i.p.) was investigated by determining the STLs to move into the dark compartment. In an additional experiment, a single group of seven rats received two injections; the first injection contained the most effective dose of DL77 and was administered 30–45 min before the PAP test, and the second injection contained RAMH (10 mg/kg, i.p.) which was administered 15–20 min prior PAP test. The CNS penetrant H3R agonist RAMH was injected 15–20 min before the start of test conduction to ensure its presence in the CNS, as RAMH shows fast metabolism (Krause et al., 2001). Doses of DL77, DOZ, and RAMH were chosen according to previously published results in the same species of rodents (Orsetti et al., 2001, 2002; Khan et al., 2015; Sadek et al., 2016a,c) (Figures 1, 2).

#### NOR test

Recognition memory was assessed in a novel object recognition (NOR) test as previously described (Ennaceur and Delacour, 1988; Izquierdo et al., 1999; de Lima et al., 2005; Karasawa et al., 2008; Alachkar et al., 2017). The experiments were conducted in a black open field box (50 × 35 × 50 cm), and the experimental procedure included two sessions of habituation of 1 h interval, whereby the animals were provided 3 min time to explore the apparatus. On the test day and after a 3 min exploration of the apparatus, two novel objects were introduced in two corners (~30 cm apart from each other). The objects (9 × 5 × 9 cm) used in this study were wood blocks and existed in different shape and color, but were of the same size. They appeared devoid of natural significance for the animals and had never been linked with reinforcement. They were adequately heavy to be not displaced by the tested rats. The experimental session consisted of two trials T1 and T2, each lasting for 3 min. In T1, rats were exposed to two identical objects, and those exploring the objects for less than 10 s during T1 were excluded from the tests. In T2, performed 120 min (for STM) or 24 h (for LTM) later, rats were exposed to two objects, one of which was a duplicate of familiar object in order to exclude olfactory traits, and another novel object. The role (familiar or novel object) as well as the relative position of the two objects were counterbalanced and arbitrarily permuted during T2. The principle measure was the time spent by the rat for exploring objects during both trials, namely T1 and T2. The test box as well as all used objects were thoroughly cleaned with 70% (volume/volume; v/v) alcoholic solution. All sessions of NOR test were executed during the light phase (8:00–12:00 a.m.). In order to detect procognitive effects of test compound, MK801 and test compound were dissolved in isotonic saline and injected i.p. at a volume of 1 ml/kg 30–45 min. following T1. The control groups received an equivalent volume of saline injection. The choice of doses and pretreatment times for each compound was decided according to the results of most promising dose in PAP and was derived from previously reported procognitive studies (Bernaerts et al., 2004; da Silva et al., 2009; Goshadrou et al., 2013; Khan et al., 2015; Sadek et al., 2016c; Alachkar et al., 2017; Sultan et al., 2017).

##### Dose regimen

**Figure 3:**
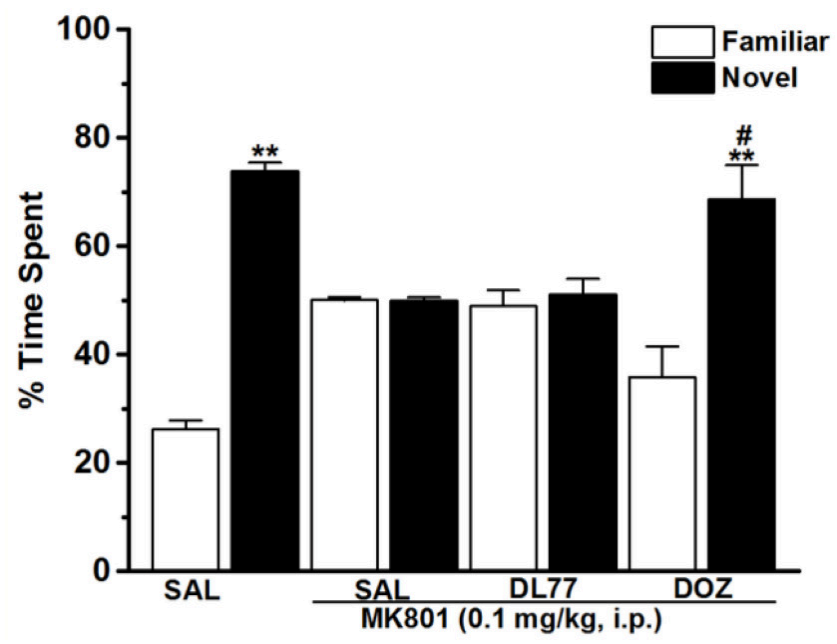
Effects of DL77 on MK801-induced STM cognitive deficits in NOR paradigm in rats. Following training session T1, DL77 (5 mg/kg) or DOZ (1 mg/kg) was administrated i.p., followed 30 min later by i.p. administration of MK801 at a dose of 0.1 mg/kg. The test session T2 was performed 120 min (STM) after the training session T1. Results are calculated as individual percentage of time spent exploring familiar (white columns) and novel (black columns) objects. Data represent mean ± SEM (*n* = 6). ^**^P < 0.001 vs. respective familiar object. ^#^*P* < 0.05 vs. MK801-treated group.

**Figure 4:**
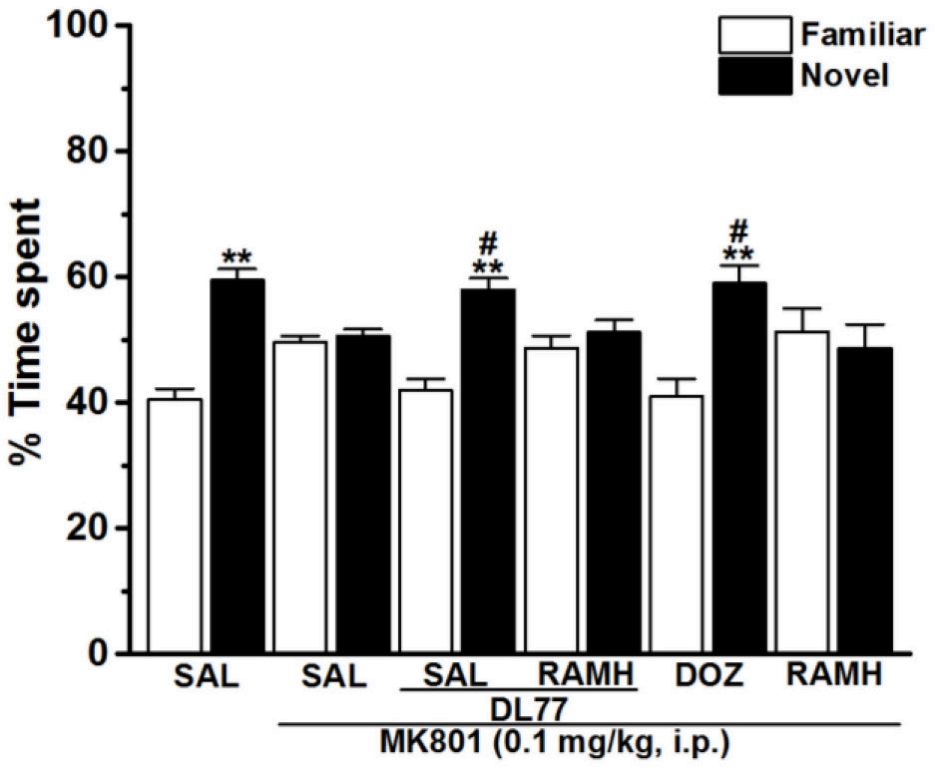
Effects of DL77 on MK801-induced LTM cognitive deficits in NOR paradigm in rats. Following training session T1, DL77 (5 mg/kg) or DOZ (1 mg/kg) was administrated i.p., followed 30 min later by i.p. injection of MK801 at a dose of 0.1 mg/kg. The test session T2 was performed 24 h (LTM) after the training session T1. Results are calculated as individual percentage of time spent exploring familiar (white columns) and novel (black columns) objects. Data represent mean ±SEM (*n* = 6). ^**^P < 0.001 vs. respective familiar object. ^#^*P* < 0.05 vs. MK801-treated group.

Six groups of six to eight rats each were used for the detection of a procognitive effect in STM. The groups were injected with Saline, MK801, MK801+DL77 (5 mg/kg, i.p.), MK801+DL77(5 mg/kg)+RAMH (10 mg/kg, i.p.), MK801+DOZ (1 mg/kg, i.p.), or MK801+RAMH (10 mg/kg, i.p.) 30–45 min before T2, respectively, and the counteracting effects of DL77 on cognitive deficits induced with MK801 were assessed by measuring the time spent by the rat in exploring objects in both trials T1 and T2 for STM (Figure 3). In an additional experiment, the procognitive effect of DL77 on MK801-induced memory impairments was confirmed by abrogative study in which the respective promising dose (5 mg/kg, i.p.) of DL77 and RAMH (10 mg/kg, i.p.) were co-administered to a separate group of six rats 30–45 min prior to T2 and 24 h after T1. The latter experiment was carried out to confirm the procognitive effect for LTM, whereas control groups received comparable saline injections (Figure 4). Doses of MK801, RAMH, and DOZ were chosen according to previous experimental protocols of NOR (Bernaerts et al., 2004; de Lima et al., 2005; da Silva et al., 2009; Goshadrou et al., 2013; Khan et al., 2015; Sadek et al., 2016c; Alachkar et al., 2017; Sultan et al., 2017).

#### EPM test

Anxiety-like behaviors were evaluated in an EPM as previously described (Jiang et al., 2016; Alachkar et al., 2017). The EPM apparatus consisted of several parts including one central part (8 × 8 cm), two opposing open and closed arms (30 × 8 cm), and nontransparent walls (30 cm in height). Between every session, both the plat form and the wall were thoroughly cleaned using 10% alcoholic spray. Animals were placed individually in the center arena of the maze (50 cm above the floor) facing an open arm, and test sessions took place in the light phase (9:00–12:00 a.m.). The amount of time spent with head and forepaws on the open arms and closed arms of the maze as well as the number of entries into each arm was manually scored for a session of 5 min. The maze was thoroughly cleaned between sessions using a tissue dampened with 70% (volume/volume; v/v) alcohol to remove the odor after each rat was tested. The total number of entries into the closed arms is usually used as an index of locomotor activity in the test.

##### Dose regimen

**Figure 5:**
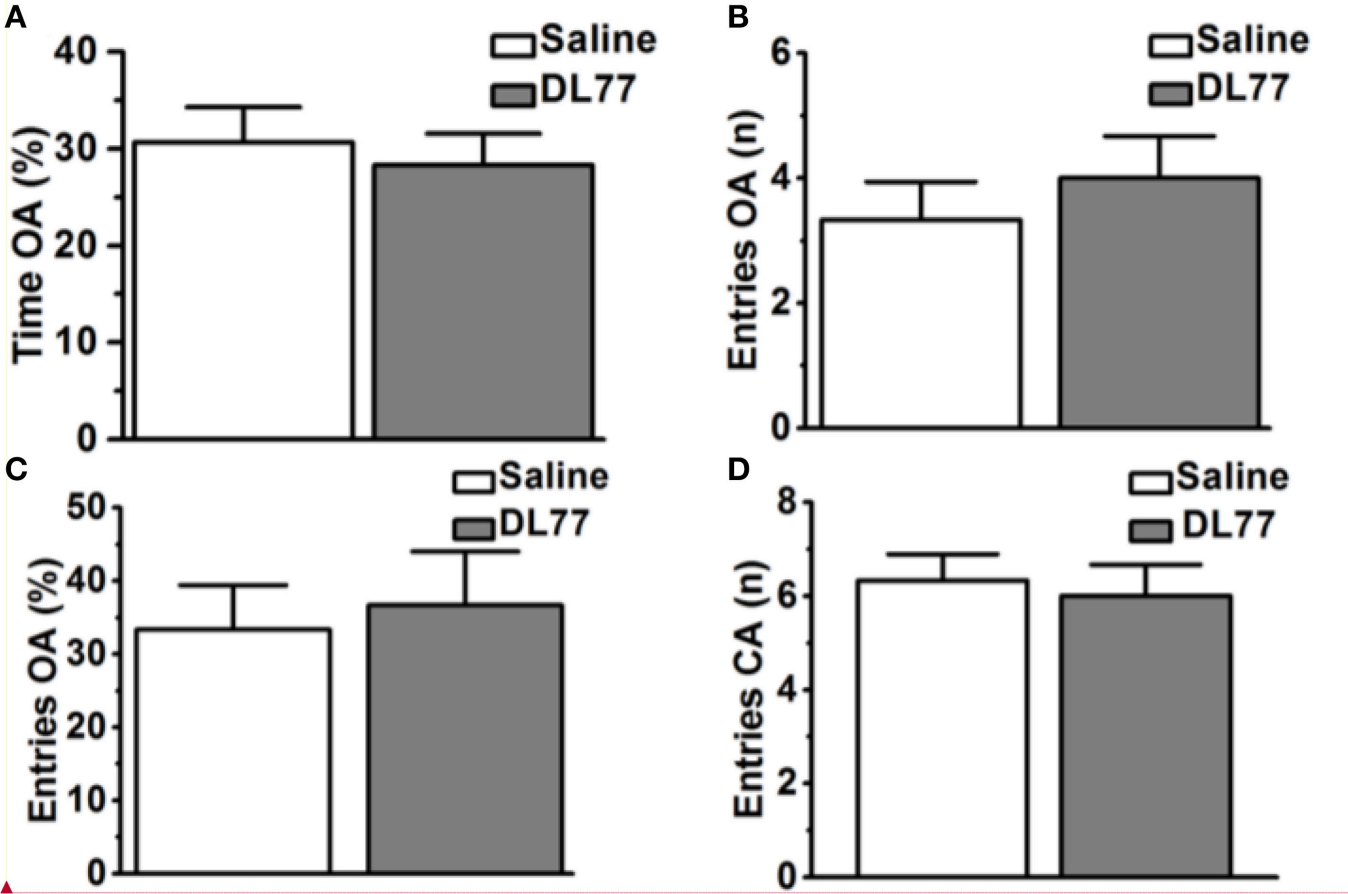
Effects of acute systemic administration of DL77 on exploratory behavior in EPM. DL77 (5 mg/kg) did not alter the percentage of time spent on the open arms of the EPM **(A)**, increase the number of entries into the open arms **(B)** and the percentage of entries into the open arms **(C)**. Acute systemic pretreatment with the H3R antagonist/inverse agonist DL77 (5 mg/kg, i.p.) did not affect the number of closed arm entries **(D)**. Data are expressed as mean ± SEM (*n* = 6).

Rats were divided into two groups of six rats each. One group received saline injection i.p. 30–45 min before the test and test group received the H3R antagonist DL77 (5 mg/kg, i.p.) for testing its modulating effects on anxiety and locomotion (Figure 5).

### Statistical analysis

IBM® SPSS Statistics® version 24 software (IBM Middle East, Dubai, United Arab Emirates) was used for all statistical comparisons. The results of NOR were expressed as the means and standard errors (SEM) of the exploratory time spent by the rat exploring both objects in T1 and T2. The results of the EPM test were expressed as the means and SEM of the percentage time spent on open arms, number of entries into the open arms, the percentage of entries into the open arms, and number of entries into closed arms. Results of NOR and EPM were analyzed by using a two-way analysis of variance (ANOVA). When relevant *post-hoc* comparisons were performed with *Bonferroni's* test in case of a significant main effect. STLs observed in PAP test were expressed as means and SEM. Because of the arbitrary cutoff latency used, the results were evaluated by using nonparametric Kruskal–Wallis ANOVA, and the differences between groups were estimated by individual Mann–Whitney *U*-tests. The criterion for statistical significance was set at *P*-value of < 0.05.

## Results

### Memory-enhancing effects of H3R antagonist DL77 and standard drug DOZ on memory deficits induced with MK801 in PAP task

The effect of acute systemic injection of DL77 at three different doses, namely 2.5, 5, and 10 mg/kg, and DOZ (1 mg/kg) on memory deficits induced with MK801 in an inhibitory PAP test in rats are shown in Figure 1. Statistical analysis of observed results indicated that acute systemic pretreatment with the three doses of DL77, and DOZ (1 mg/kg) prior to retention test exhibited a significant memory-enhancing effect on STLs [*H*_(5)_ = 28.29; *P* < 0.001; Figure 1]. As shown following pairwise comparisons, MK801 (0.1 mg/kg) decreased STL time when compared to the (saline)-treated control group with (*U* = 28.00, *P* < 0.05). Moreover, DL77 tested in three different doses (2.5, 5, and 10 mg/kg) showed significant improving effect on STLs time when compared to (MK801)-treated group with (all *P* < 0.05). However, pretreatment with DL77 (5 mg/kg, i.p.) was found to be not significantly different from (Saline)-treated control rats (*U* = 38.50, *p* = 0.073) (Figure 1). Furthermore, DL77 (5 mg/kg) showed signicantly higher improving effects on STL when compared with the DOZ(1 mg)-provided memory-enhancing efects with (*U* = 5.00, *P* < 0.05) (Figure 1).

### Abrogative effects of RAMH on the memory improvement provided with DL77 in MK801-induced deficits in PAP task

For this experiment, a group of seven animals was injected with the most promising dose of DL77 (5 mg/kg, i.p.) 30–45 min prior to test, and also adminstered with the H3R agonist RAMH (10 mg/kg, i.p.) 15 min before the test session (Figure 2). As shown in Figure 2, statistical analysis revealed that this factor had a significant effect on the STL time [*H*_(5)_ = 31.32; *P* < 0.001]. Moreover, pairwise comparisons indicated that acute systemic pretreatment with DL77 (5 mg/kg, i.p.) enhanced STL time when compared to the (MK801)-amnesic group with (*U* = 49.00; *P* < 0.05). Interestingly, the improvmemnt of observed STL time provided with DL77 was abrogated following acute co-injection of RAMH (*U* = 49.00; *p* = 0.701: MK801-amnesic group vs. MK801+DL77+RAMH group, Figure 2). Noteably, acute systemic administration of RAMH (10 mg/kg, i.p.) alone failed to affect the observed STL time in MK801-amnesic group as well as in Saline group with no significant differences (*U* = 30.50; *p* = 0.442) and (*U* = 20.50; *p* = 0.620), respectively (Figure 2).

### Modulating effects of H3R antagonist DL77 and DOZ on the STM deficits induced with MK801 in NOR task

The results observed for the total time exploring both objects during T1 and T2 were not significantly different when comparing groups pretreated with saline and those injected with MK801 (Table 1). The latter experimental observation is substantial to exclude any confounding factors, e.g., that the post-training treatment with the amnesic compound MK801 in the first experiment did not modify sensorimotor considerations such as locomotor activity and motivations of tested animals. Furthermore, statistical results revealed that no significant differences were present in exploratory times between the two identical objects during T1 for each respective experimental group of animals (Table 1). The observed results, also, showed that acute systemic pretreatment with DL77 (5 mg/kg, i.p.) and standard drug DOZ (1 mg/kg, i.p.) significantly counteracted time spent exploring objects in T2 with [*F*_(3, 20)_ = 13.76; *P* < 0.001] when injected 30–45 min after T1 (Figure 3). As revealed by conducted post hoc analyses, MK801 (0.1 mg/kg, i.p.) decreased memory toward the novel object in T2 when compared to the (saline)-treated group with [*F*_(1, 10)_ = 140.96; *P* < 0.001], and DOZ (1 mg/kg, i.p.) significantly counteracted this memory deficit in STM in T2 when compared to (MK801)-amnesic group [*F*_(1, 10)_ = 7.02; *P* < 0.05)]. Contrary, acute systemic administration of DL77 (5 mg/kg, i.p.) did not counteract the decreased STM when compared to (MK801)-amnesic group with [*F*_(1, 10)_ = 0.08; *p* = 0.772].

**Table 1:** Effects of DL77 on MK801-induced total exploratory time spent with both objects during training and test session in NOR in rats.

**Group**		**Time exploring objects (s)**			
	***n***	**Training session STM**	**Test session STM**	**Training session LTM**	**Test session LTM**
Saline	6	37.50 ± 3.2	41.83 ± 2.67	36.67 ± 2.93	38.17 ± 3.86
MK801	8	36.00 ± 2.73	42.25 ± 1.57	23.63 ± 2.73	28.88 ± 2.32
MK801 + DL77(5 mg/kg)	8	24.88 ± 2.73	28.17 ± 3.57	23.50 ± 1.71	23.63 ± 2.73
MK801+DL77(5 mg/kg)+RAMH(10 mg/kg)	6	ND	ND	23.63 ± 2.73	23.33 ± 4.87
MK801+DOZ(1 mg/kg)	6	24.83 ± 1.72	25.33 ± 1.88	23.00 ± 3.34	25.33 ± 1.68
MK801+RAMH(10 mg/kg)	6	ND	ND	21.67 ± 1.74	22.67 ± 2.35

*Data are expressed as mean ±SEM of 6 or 8 animals per experimental group. There were no significant differences in total exploratory times among treated groups*.

### Modulating effects of H3R antagonist DL77 and DOZ on the LTM deficits induced with MK801 in NOR task

The results showed that H3R antagonist DL77 (5 mg/kg, i.p.) and standard drug DOZ (1 mg/kg, i.p.) when injected 30–45 min before T2 exhibited a significant counteracting effect on time spent exploring objects in T2 with [*F*_(5, 30)_ = 2.67; *P* < 0.05] (Figure 4). Moreover, subsequent *post-hoc* analyses revealed that MK801 (0.1 mg/kg, i.p.) decreased memory for the novel object in T2 when compared to the (MK801)-amnesic group with [*F*_(1, 10)_ = 13.92; *P* < 0.05]. However, acute systemic administration with the H3R antagonist DL77 (5 mg/kg, i.p.) significantly counteracted the induced memory deficits in LTM when compared to (MK801)-amnesic group with [*F*_(1, 10)_ = 9.05; *P* < 0.05] (Figure 4). Moreover, the LTM-enhancing procognitive effect provided by DL77 (5 mg/kg, i.p.) was reversed when rats were co-administered with RAMH (10 mg/kg, i.p., i.p.) as compared to the (MK801)-amnesic group with [*F*_(1, 10)_ = 0.03; *p* = 0.877]. Notably, the significant LTM enhancing effect provided with DL77 (5 mg/kg, i.p.) was comparable to the effects observed by the standard drug DOZ (1 mg/kg, i.p.) in T2 with [*F*_(1, 10)_ = 0.08; *p* = 0.788]. Also and similar to the results observed in STM, statistical analyses revealed that RAMH (10 mg/kg, i.p.) alone did not alter LTM in T2 when compared to the MK801-amnesic group with [*F*_(1, 10)_ = 0.20; *p* = 0.67] (Figure 4).

### Effect of DL77 on rat anxiety and locomotor activity in EPM test

Figure 5 shows the observed effects of acute systemic injection of Saline or H3R antagonist DL77 (5 mg/kg, i.p.) on the anxiety parameters of rats exposed to the EPM, namely the percentage of time spent in open arms, the number of entries into open arms, the percentage entries into open arms, and locomotor activity expressed as the number of entries into closed arms. Subsequent *post-hoc* analyses showed that DL77 (5 mg/kg, i.p.) did not alter the percentage of time spent exploring the open arms of the maze during a 5 min session when compared to saline-treated group with [*F*_(1, 10)_ = 0.19, *p* = 0.67] (Figure 5A). Moreover, further analyses of data describing the number and percentage of entries into the open arms of the maze [*F*_(1, 10)_ = 0.45, *p* = 0.52; *F*_(1, 10)_ = 0.10, *p* = 0.76, respectively] yielded practically the same results. As depicted in Figures 5B,C, no significant differences were obtained between the results in the DL77(5 mg/kg)-treated group and those observed in the saline-treated group (Figures 5B,C). Interestingly, the number of closed arm entries following DL77 injection was not significantly changed with [*F*_(1, 10)_ = 0.12, *p* = 0.73], demonstrating that locomotor activity as such was not modulated following acute systemic administration with the H3R antagonist DL77 (5 mg/kg, i.p.) (Figure 5D).

## Discussion

In the current series of experiments, acute systemic injection of 2.5, 5, and 10 mg/kg of DL77 ameliorated the memory deficits induced by MK801 in an inhibitory PAP in rats. The observed results revealed that DL77 significantly reversed the memory deficits induced by MK801 (Figure 1). Since MK801 is a very well-known NMDA receptor antagonist and NMDA receptors were confirmed with their important role in both consolidation and retrieval processes, it is likely that DL77 partially counteracted memory deficits induced with MK801 through direct interaction and activation of NMDA receptors by the increased release of central histamine as a consequence of antagonistic activity of DL77 at histamine H3-auto-receptors. These latter results are in agreement with earlier studies in which histamine enhanced NMDA receptor-mediated neurotransmission in cultured hippocampal cells, indicating that the interaction between histamine and NMDA receptors might facilitate the histamine's capability to counteract MK801-induced amnesic effect (Vorobjev et al., 1993; Xu et al., 2005; Brabant et al., 2013; Sadek et al., 2016a). Notably, the procognitive effect provided by DL77 was dose-dependent, as DL77 at a dose of 5 mg/kg provided significantly higher counteracting effect on decreased STL time when compared to the lower as well as higher dose (2.5 and 10 mg/kg), respectively, indicating that an optimum of DL77-provided memory-enhancing effect might have been reached with a dose of 5 mg/kg, and that off-targets effects could have been present following acute systemic administration of DL77 at a dose of 10 mg/kg (Figure 1). Interestingly, the latter observations for the dose dependency are, also, similar to those observed in previous preclinical experiments in rodents (Benetti and Izquierdo, 2013; Benetti et al., 2013; Sadek et al., 2016c). Moreover, the results observed in regard to dose dependency strongly support our previous results detected for the effects of H3R antagonist DL77 (2.5, 5, and 10 mg/kg, i.p.) on different memory stages, namely acquisition, consolidation, and retrieval (Sadek et al., 2016c). Notably, the observed procognitive effects for DL77 (5 mg/kg) were comparable to those obtained for the reference drug DOZ, a procognitive compound available for memory-enhancing effect, since there is up to date no reference drug which is targeting H3Rs (Figure 1). Moreover, the procognitive effects found for DL77 (5 mg/kg) were completely reversed when animals were pretreated with the CNS penetrant H3R agonist RAMH, indicating clearly that blockade of H3Rs substantially contributes in the central neurotransmissions associated with retrieval processes of tested animals (Figure 2). Unlike the inhibitory PAP, the NOR paradigm in rodents does not involve a reward or a punishment, and it takes advantage of their innate interest for exploring their environment, as it is established on the natural behavior of rodents. Therefore, the behavioral reaction of tested animals is not biased by reinforcement/response interactions of tested rats. Also, the NOR task is a behavioral paradigm used in animal models to evaluate aspects related to cognitive performance, e.g., recognition memory (Jaaro-Peled, 2009; Tseng et al., 2009; Brown et al., 2013; Callahan et al., 2017). Furthermore, previous preclinical studies revealed that NOR paradigm can be utilized in cognitive related experiments due to its sensitivity to both agents capable of impairing (Ennaceur and Delacour, 1988; Ennaceur and Meliani, 1992a,b) as well as enhancing cognition (Lebrun et al., 2000; Barak and Weiner, 2011) following acute systemic pre- and/or post-training administration of the individual agent (King et al., 2004; de Lima et al., 2005; Pichat et al., 2007). In the current study, acute systemic post-training injection of DL77 (5 mg/kg, the most promising dose in PAP test) significantly improved the exploratory time spent with the novel object compared with the familiar objects (Figure 3). These observations are in consensus with previous reports revealing that various H3R antagonists belonging to the imidazole-based class, e.g., thioperamide and clobenpropit (Giovannini et al., 1999), and to the non-imidazole-based class, e.g., pitolisant (Ligneau et al., 2007); GSK189254 (Giannoni et al., 2010); SAR110894 (Griebel et al., 2012), and ABT-239 (Provensi et al., 2016) counteracted the memory-impairing effects of MK801 and scopolamine in NOR tests using different rodents. In our conducted experiments, DL77 potently counteracted the LTM-impairing effects induced with MK801, and these DL77-provided effects were entirely reversed when rats were co-injected with the H3R agonist RAMH (Figure 3 and Table 1). The latter observations are in agreement with an earlier study in which RAMH abolished the memory-enhancing effects provided by H3R antagonist ciproxifan on LTM (Pascoli et al., 2009). Unlike the results observed for DL77 on LTM, acute systemic post-training administration of DL77 did not increase the exploratory time spent with the novel objects in STM when compared with the familiar objects (Figure 4 and Table 1). The latter results are in discrepancy with earlier studies in which H3R antagonist ABT-239 enhanced STM in mice (Provensi et al., 2016). The discrepancy in the results observed in STM might be explained with the different species used or the differences in doses used or in the conduct of experiments. Accordingly, acute systemic post-training administration of MK801 was used in the current study to induce amnesia, whereas natural memory decline as well as presence or absence of histaminergic neurotransmission were examined in the study conducted by Provensi et al. (2016). Moreover, the current experimental findings in NOR obviously point toward profound contribution of histaminergic H3Rs in neuronal circuits associated with the DL77-provided procognitive effects in LTM (Figure 4 and Table 1). The lack of memory-enhancing activity of DL77 in STM is in agreement with previous reports in which no differences were found in time spent exploring novel object in wild type (intact brain histamine) and histidine decarboxylase-knocked out mice (lack of brain histamine) when tested 2 h post-training (STM), but not when testing 24 h post-training (LTM), indicating that histaminergic neurotransmission is more involved the neural circuits which modulate the LTM (Acevedo et al., 2006, 2007; Provensi et al., 2016). Interestingly, several H3R antagonists have in earlier preclinical studies been designated as talented candidates for AD and were suggested to be of possible novel therapeutics due to their capability to interact with H3 auto- and hetero-receptors, modulating the synthesis and release of numerous brain neurotransmitters critical for cognition, including histamine, dopamine, and acetylcholine (Brioni et al., 2011; Sadek and Stark, 2015; Sadek et al., 2016b). The EPM test is considered to be one of the most used animal tests in neuroscience to assess emotionality-related behaviors, e.g., anxiety, based on the innate tendency of animals to avoid open spaces in favor of protected areas, while measuring percent and/or number of closed arms entries reportedly ensures that behavior observed in the maze did not simply reflect drug-induced alterations in locomotor activity (Fernandes and File, 1996; Hogg, 1996; Alachkar et al., 2017). Notably, DL77 at the dose (5 mg/kg) that exhibited the most encouraging procognitive effect in PAP and NOR paradigms did not affect anxiety levels of the adult male Wistar rats (Figures 5A–D). Also, DL77 administered at the same dose (5 mg/kg) did not affect the number of closed arm entries, indicating that DL77 failed to modify locomotion of tested rats, demonstrating that enhanced memory performance in PAP as well as NOR is not related to modified emotional responses or altered spontaneous locomotor activity considered as confounding factors when assessing memory-enhancing effects in PAP and NOR (Figure 5D) (McGaugh and Roozendaal, 2009; Charlier et al., 2013). The latter results are, also, in line with our earlier results in which acute systemic injection of DL77 (2.5, 5, and 10 mg/kg, i.p.) did not affect spontaneous locomotion of the same animal species when tested in the open field task (Sadek et al., 2016c). Therefore, it is unlikely that acute systemic injection of DL77 (5 mg/kg, i.p.) in the post-training sessions provided memory-enhancing effects in PAP and NOR paradigms due to a nonspecific effect rather than improved learning tasks conducted in the training sessions of both paradigms.

## Conclusion

**Figure 6:**
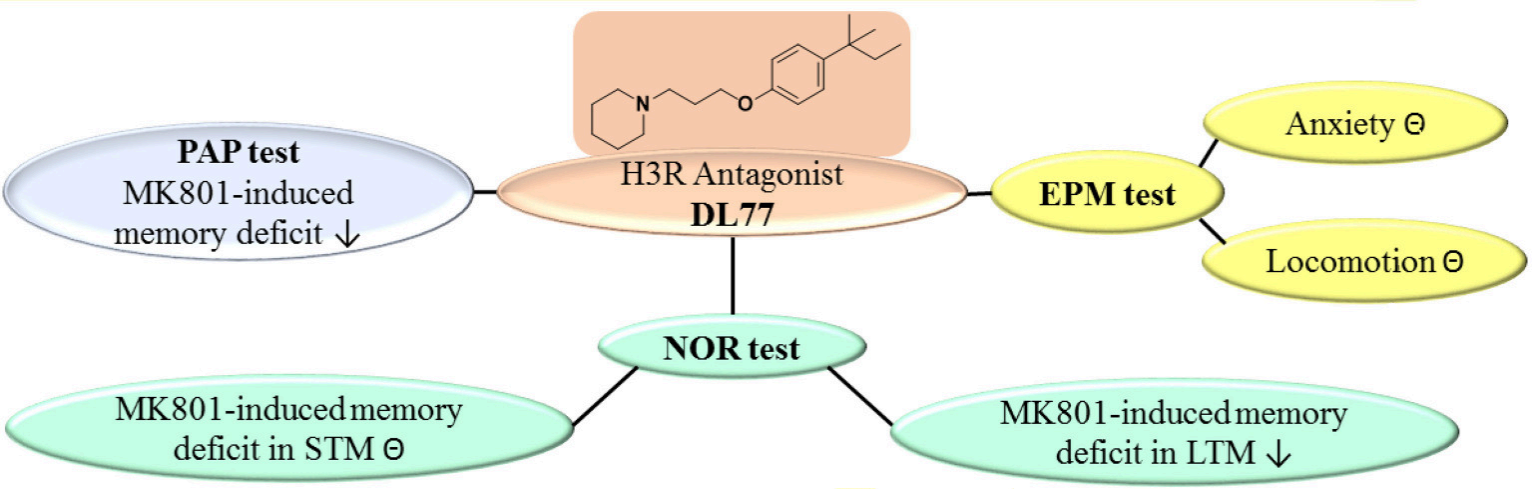
Schematic representation of proposed ameliorative effects for H3R antagonist DL77 in MK801-induced memory deficit in PAP, NOR, and EPM. PAP, passive avoidance paradigm; NOR, novel object recognition; EPM, elevated plus maze; **⊝**, no effect; ↓, ameliorates.

The results show that the non-imidazole H3R antagonist DL77 ameliorated cognitive deficits induced by the NMDA receptor antagonist MK801 in an inhibitory PAP and in NOR paradigms in rats (Figure 6). Moreover, the results observed in PAP as well as LTM of NOR indicated that DL77 ameliorated cognitive deficits through blockade of H3Rs, demonstrating the therapeutic prospective of H3R antagonists in the future treatment of neurodegenerative diseases, e.g., AD. However, additional preclinical experiments in other behavioral test models and with several rodent species are still warranted to comprehend the translational validity of the prospective use of H3R antagonists in future therapy of neurodegenerative diseases.

## Author contributions

BS was responsible for the study concept, design, acquisition, and analysis of animal data; NK provided technical support to the conducted behavioral experiments; NE and NK conducted behavioral experiments; KK-K and DŁ were responsible for the generation, synthesis, and pharmacological *in vitro* characterization the H3R antagonist DL77; BS and NE drafted the manuscript; KK-K, DŁ, and SO provided critical revision for the manuscript; All authors critically reviewed content and approved final version for publication.

### Conflict of interest statement

The authors declare that the research was conducted in the absence of any commercial or financial relationships that could be construed as a potential conflict of interest.

## Abbreviations

AD: Alzheimer disease
H3Rs: histamine H3 receptors
RAMH: *R*-(α)-methyl-histamine
DOZ: donepezil
MK801: dizocilpine
PAP: passive avoidance paradigm
STL: step-through latency
NOR: novel object recognition
STM: short-term memory
LTM: long-term memory
EPM: elevated plus maze
p.o.: per oral
i.p.: intraperitoneal.
